# *catena*-Poly[lead(II)-bis­(μ-2-amino-1,3-benzothia­zole-6-carboxyl­ato)]

**DOI:** 10.1107/S1600536810049330

**Published:** 2010-11-30

**Authors:** Ke-Ke Zhang, Xin Fang, Hai-Yang Yu, Hua Ke, Jun-Dong Wang

**Affiliations:** a College of Chemistry and Chemical Engineering, Fuzhou University, Fuzhou, Fujian 350108, People’s Republic of China

## Abstract

The title complex, [Pb(C_8_H_5_N_2_O_2_S)_2_]_*n*_, consists of one Pb^II^ ion located on a crystallographic twofold axis and two symmetry-related 2-amino-1,3-benzothia­zole-6-carboxyl­ate (ABTC) ligands. The central Pb^II^ ion has a (4 + 2) coordination by four O atoms of the two ABTC ligands and two weaker Pb—S bonding inter­actions (Pb—S secondary bonds) from S atoms of other two neighbouring ABTC ligands. These bonds link the metal ions into zigzag chains along the *c* axis, which, in turn, aggregate through π–π inter­actions [centroid–centroid distance = 3.7436 Å] between ABTC rings and N—H⋯O and N—H⋯N hydrogen bonds.

## Related literature

For applications of benzothia­zole and its derivatives, see: Petkova *et al.* (2000 ▶); Leng *et al.* (2001 ▶); Karlsson *et al.* (2003 ▶); Ćaleta *et al.* (2009 ▶); Tzanopoulou *et al.* (2010 ▶).  For the use of benzothia­zoles in building novel complexes, see: Vuoti *et al.* (2007 ▶); Zou *et al.* (2004 ▶); Ng *et al.* (2008 ▶); Chen *et al.* (2010 ▶); For our recent work on the design and synthesis of benzothia­zole derivatives, see: Fang *et al.* (2010 ▶); Lei *et al.* (2010 ▶). For secondary Pb—S bonds, see: Chan & Rossi (1997 ▶); Turner *et al.* (2008 ▶). For van der Waals radii, see: Bondi (1964 ▶). For (4 + 2) coordination, see: Chan & Rossi (1997 ▶); Calatayud *et al.* (2007 ▶); Turner *et al.* (2008 ▶); Pena-Hueso *et al.* (2008 ▶). For π–π inter­actions, see: Sredojević *et al.* (2010 ▶). For the preparation of the 2-amino­benzothia­zole-6-carb­oxy­lic acid ligand, see: Das *et al.* (2003 ▶). For a description of the Cambridge Structural Database, see: Allen (2002 ▶). 
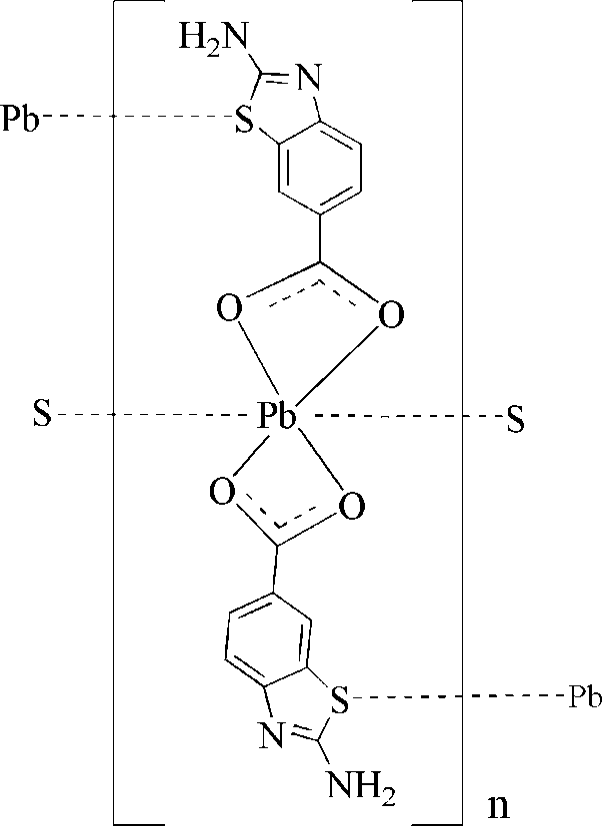


## Experimental

### 

#### Crystal data

[Pb(C_8_H_5_N_2_O_2_S)_2_]
*M* *_r_* = 593.59
Monoclinic, 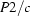

*a* = 10.909 (2) Å
*b* = 4.8271 (10) Å
*c* = 15.980 (3) Å
β = 100.02 (3)°
*V* = 828.6 (3) Å^3^
*Z* = 2
Mo *K*α radiation
μ = 10.47 mm^−1^
*T* = 293 K
0.39 × 0.29 × 0.15 mm

#### Data collection

Rigaku Saturn 724 CCD area-detector diffractometer
Absorption correction: numerical (*NUMABS*; Higashi, 2000) ▶ *T*_min_ = 0.378, *T*_max_ = 1.000
6088 measured reflections
1890 independent reflections
1871 reflections with *I* > 2σ(*I*)
*R*_int_ = 0.075

#### Refinement

*R*[*F* ^2^ > 2σ(*F* ^2^)] = 0.037
*wR*(*F* ^2^) = 0.096
*S* = 1.11
1890 reflections
123 parameters
H-atom parameters constrained
Δρ_max_ = 2.13 e Å^−3^
Δρ_min_ = −2.56 e Å^−3^

### 

Data collection: *CrystalClear* (Rigaku, 2007 ▶); cell refinement: *CrystalClear*; data reduction: *CrystalClear*; program(s) used to solve structure: *SHELXS97* (Sheldrick, 2008 ▶); program(s) used to refine structure: *SHELXL97* (Sheldrick, 2008 ▶); molecular graphics: *ORTEX* (McArdle, 1995 ▶); software used to prepare material for publication: *SHELXL97*.

## Supplementary Material

Crystal structure: contains datablocks global, I. DOI: 10.1107/S1600536810049330/bg2378sup1.cif

Structure factors: contains datablocks I. DOI: 10.1107/S1600536810049330/bg2378Isup2.hkl

Additional supplementary materials:  crystallographic information; 3D view; checkCIF report

## Figures and Tables

**Table 1:** Hydrogen-bond geometry (Å, °)

*D*—H⋯*A*	*D*—H	H⋯*A*	*D*⋯*A*	*D*—H⋯*A*
N1—H1*B*⋯O1^i^	0.86	2.11	2.973 (7)	179
N1—H1*A*⋯N2^ii^	0.86	2.09	2.934 (7)	168

Symmetry codes: (i) 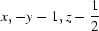; (ii) 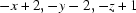.

**Fig. 1.**
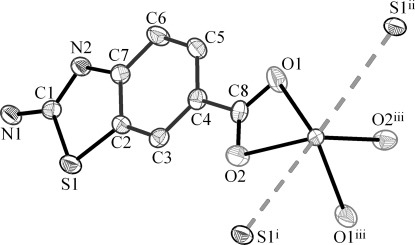
The crystal structure of (I),drawn with 40% probability displacement ellipsoids. H atoms have been omitted for clarify. Symmetry codes: (i) 1 - x, -y,1 - z; (ii)x,-y,1/2 + z; (iii)1 - x, y, 3/2 - z.

**Fig. 2.**
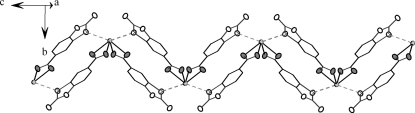
A view of the one-dimensional chain formed by Pb—S secondary bonds in (I).All H atoms have been omitted and all C atoms are shown as wires or sticks for clarify.

**Fig. 3.**
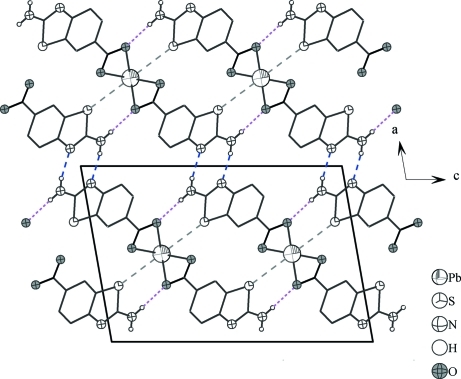
A packing diagram for (I), showing some of the hydrogen bonds (blue and red dashed lines) and π-π interactions along the b direction. Most of the H atoms have been omitted except those involved in the weak interactions. All C atoms are shown as wires or sticks for clarify.

## supplementary crystallographic information

### Comment

In recent years, benzothiazole and its derivatives have been attracting more attention because they exhibit interesting optical and biological activities, which made them widely used in many fields, such as fluorescent materials, nonlinear optical materials, pesticides, anti-tumor and anti-microbial drugs, *etc*. (Petkova *et al.*, 2000; Leng *et al.*, 2001; Karlsson *et al.*, 2003; Ćaleta *et al.*, 2009). Related structural studies are partly focused on the fact that the benzothiazole ring contains N, S and O as potential donor atoms, which exhibit good coordination capacity, and so are propitious to build novel complexes (Zou *et al.*,2004; Vuoti *et al.*, 2007; Ng *et al.*, 2008; Chen *et al.*,2010;). By reviewing their metal complexes (Cambridge Structural Datebase, Version of 5.31 of August 2010; Allen, 2002), it was found that most metal atoms only match with N atom of thiazole ring, but not the S atom (because the coordination capacity of S is much weaker than N), as long as these metal atoms have interaction with the thiazole ring. In our recent work, accompanied with the design and synthesis of benzothiazole derivatives (Lei *et al.*, 2010; Fang *et al.*, 2010), complexes of benzothiazole derivatives with metal atoms were composed and structurally analyzed to explore their coordination behaviors. In this paper, we report the structure of a coordination polymer of lead and 2-amino-1,3-benzothiazole-6-carboxylate ligand (ABTC), where the coordination mode of S with Pb is seen as a secondary Pb—S bond (Chan *et al.*, 1997; Turner *et al.*, 2008).

The asymmetric unit of the complex contains a Pb^II^ ion located on a two fold axis and one independent 2-amino-1,3-benzothiazole-6-carboxylate (ABTC) ligand (Figure 1). The central Pb^II^ ion is coordinated by four O atoms of two ABTC ligands in a pyramid fashion with the Pb^II^ ion at the apex, covalently bounded to the four O atoms making up the base of the pyramid. The four Pb—O bonds are Pb1—O1 and Pb1—O1^iii^, (iii): -*x*+1, -*y*, -*z*+1, with a distance of 2.395 (5) Å, and Pb1—O2 and Pb1—O2^ii^, (ii) -*x*+1, *y*, -*z*+3/2; with a distance of 2.366 (4) Å. The stereochemistry of the distorted pyramid is described by angles of O1—Pb—O1^iii^, 106.4 (3)^°^, and O2—Pb—O2^iii^, 102.8 (3)^°^, and the sides of the base defined by O1—O2 and O1^iii^—O2^iii^, distanced 2.1708 (60) Å, and O1—O2^iii ^and O2—O1^iii^, distanced 3.081 (7) Å .

In the crystal, two S atoms also interacte with the apical Pb^II^ ion with so-called secondary bonds, where the Pb—S distance [Pb1—S1^i^ ( (i) *x*, -*y*, *z*+1/2) and Pb1—S1^ii^, with a distance of 3.3894 (17) Å] is shorter than the corresponding sum of the van der Waals radii (3.80 Å) of Pb and S atoms (Bondi, 1964). So the Pb^II^ ion in this structure should be described as (4 + 2) coordinated (Chan *et al.*,1997; Calatayud *et al.*,2007; Turner *et al.*, 2008; Pena-Hueso *et al.*, 2008). Under this coordination mode, each ABTC ligand acts as a linear linker to coordinate two metal centers, while each metal ion is linked to four ABTC ligands, then, along the *c* axis, one-dimensional zigzag chains are formed (Figure 2).

Along the *b* axis, neighboring chains are linked by N—H···O H-bonds and *π-π* interactions between the thiazole and benzene rings [with perpendicular distance of 3.4184Å and centroid-centroid distance of 3.7436 Å]. Simultaneously, there is an interaction between the benzene ring and the carboxyl group coordinated on the Pb^II^ ion, described by the 4-membered ring of O1—C8—O2—Pb1, with a perpendicular distance of 3.5021Å and centroid-centroid distance of 3.5740 Å (Sredojević *et al.*,2010).

Finally, along the *a* axis, neighboring chains are further connected to each other by N—H··· N hydrogen bonds which complete an infinite three-dimensional framework of the structure (Table 1 and Figure 3).

It is worth noting that S secondary bonds were also present in the previously reported complex of Ag and a benzothiazole derivative (Zou *et al.*, 2004) through the weak interaction between Ag and the S atom of the thiozole ring. Also here these secondary Ag—S bonds play an important role in building the crystal framework, cooperating with the hydrogen bonds and *π-π* interactions to build the supramolecular structure.

### Experimental

The 2-aminobenzothiazole-6-carboxylic acid ligand was obtained by hydrolyzing ethyl 2-amino-1,3-benzothiazole-6-carboxylate (Das *et al.* 2003). The mixture of lead acetate (0.0379 g, 0.10 mmol), 2-aminobenzothiazole-6-carboxylic acid (0.0194 g, 0.1 mmol), and H_2_O (5 ml) was sealed in a 15 ml stainless-steel reactor with Teflon liner and heated (10°C per hour) from room temperature to 140°C and kept at 140°C for 96 h, then cooled to room temperature again at a similar rate. Brown crystals suitable for X-ray diffraction analysis were obtained.

### Refinement

All H atoms bound to C and N atoms were located in difference Fourier syntheses and were refined as riding, with C—H distances of 0.93 Å and and N—H distances of 0.86 Å . All *U*_ iso_(H) were kept at 1.2*U*_eq_(Host).

### Crystal data

**Table:**

[Pb(C_8_H_5_N_2_O_2_S)_2_]	*F*(000) = 560
*M**_r_* = 593.59	*D*_x_ = 2.379 Mg m^−^^3^
Monoclinic, *P*2/*c*	Mo *K*α radiation, λ = 0.71073 Å
Hall symbol: -P 2yc	Cell parameters from 3030 reflections
*a* = 10.909 (2) Å	θ = 3.5–27.6°
*b* = 4.8271 (10) Å	µ = 10.47 mm^−^^1^
*c* = 15.980 (3) Å	*T* = 293 K
β = 100.02 (3)°	Prism, brown
*V* = 828.6 (3) Å^3^	0.39 × 0.29 × 0.15 mm
*Z* = 2	

### Data collection

**Table:**

Rigaku Saturn 724 CCD area-detector diffractometer	1890 independent reflections
Radiation source: fine-focus sealed tube	1871 reflections with *I* > 2σ(*I*)
graphite	*R*_int_ = 0.075
Detector resolution: 28.5714 pixels mm^-1^	θ_max_ = 27.5°, θ_min_ = 3.5°
dtprofit.ref scans	*h* = −14→12
Absorption correction: numerical (*NUMABS*; Higashi, 2000)	*k* = −6→6
*T*_min_ = 0.378, *T*_max_ = 1.000	*l* = −20→20
6088 measured reflections	

### Refinement

**Table:**

Refinement on *F*^2^	Primary atom site location: structure-invariant direct methods
Least-squares matrix: full	Secondary atom site location: difference Fourier map
*R*[*F*^2^ > 2σ(*F*^2^)] = 0.037	Hydrogen site location: inferred from neighbouring sites
*wR*(*F*^2^) = 0.096	H-atom parameters constrained
*S* = 1.11	*w* = 1/[σ^2^(*F*_o_^2^) + (0.0574*P*)^2^ + 0.8091*P*] where *P* = (*F*_o_^2^ + 2*F*_c_^2^)/3
1890 reflections	(Δ/σ)_max_ < 0.001
123 parameters	Δρ_max_ = 2.13 e Å^−^^3^
0 restraints	Δρ_min_ = −2.56 e Å^−^^3^

### Special details

**Table:**

Geometry. All e.s.d.'s (except the e.s.d. in the dihedral angle between two l.s. planes) are estimated using the full covariance matrix. The cell e.s.d.'s are taken into account individually in the estimation of e.s.d.'s in distances, angles and torsion angles; correlations between e.s.d.'s in cell parameters are only used when they are defined by crystal symmetry. An approximate (isotropic) treatment of cell e.s.d.'s is used for estimating e.s.d.'s involving l.s. planes.
Refinement. Refinement of *F*^2^ against ALL reflections. The weighted *R*-factor *wR* and goodness of fit *S* are based on *F*^2^, conventional *R*-factors *R* are based on *F*, with *F* set to zero for negative *F*^2^. The threshold expression of *F*^2^ > σ(*F*^2^) is used only for calculating *R*-factors(gt) *etc*. and is not relevant to the choice of reflections for refinement. *R*-factors based on *F*^2^ are statistically about twice as large as those based on *F*, and *R*- factors based on ALL data will be even larger.

### Fractional atomic coordinates and isotropic or equivalent isotropic displacement parameters (Å^2^)

**Table:**

	*x*	*y*	*z*	*U*_iso_*/*U*_eq_	
Pb1	0.5000	0.46437 (5)	0.7500	0.02893 (13)	
S1	0.68171 (12)	−0.6475 (3)	0.43591 (9)	0.0341 (3)	
N1	0.8579 (6)	−1.0111 (11)	0.4062 (4)	0.0358 (12)	
H1A	0.9286	−1.0940	0.4177	0.043*	
H1B	0.8061	−1.0545	0.3611	0.043*	
N2	0.9003 (4)	−0.7340 (11)	0.5281 (3)	0.0314 (10)	
O1	0.6764 (4)	0.1672 (12)	0.7523 (3)	0.0456 (12)	
O2	0.5321 (4)	0.1587 (10)	0.6401 (3)	0.0400 (10)	
C1	0.8286 (5)	−0.8158 (13)	0.4580 (3)	0.0293 (10)	
C2	0.7228 (6)	−0.4520 (10)	0.5281 (4)	0.0269 (11)	
C3	0.6541 (5)	−0.2540 (12)	0.5620 (3)	0.0293 (11)	
H3	0.5754	−0.2021	0.5341	0.035*	
C4	0.7070 (5)	−0.1345 (12)	0.6396 (3)	0.0303 (11)	
C5	0.8264 (5)	−0.2125 (15)	0.6806 (4)	0.0382 (13)	
H5	0.8603	−0.1325	0.7324	0.046*	
C6	0.8940 (6)	−0.4066 (16)	0.6445 (4)	0.0401 (14)	
H6	0.9740	−0.4530	0.6712	0.048*	
C7	0.8425 (6)	−0.5332 (12)	0.5682 (4)	0.0301 (12)	
C8	0.6357 (6)	0.0758 (12)	0.6796 (4)	0.0295 (11)	

### Atomic displacement parameters (Å^2^)

**Table:**

	*U*^11^	*U*^22^	*U*^33^	*U*^12^	*U*^13^	*U*^23^
Pb1	0.03445 (19)	0.02334 (18)	0.0291 (2)	0.000	0.00589 (12)	0.000
S1	0.0295 (6)	0.0391 (8)	0.0308 (7)	0.0058 (6)	−0.0030 (5)	−0.0049 (6)
N1	0.033 (3)	0.042 (3)	0.030 (3)	0.005 (2)	−0.001 (2)	−0.010 (2)
N2	0.0270 (19)	0.036 (3)	0.030 (2)	0.0052 (19)	0.0017 (17)	−0.0053 (19)
O1	0.052 (3)	0.055 (3)	0.028 (2)	0.021 (2)	0.0025 (18)	−0.007 (2)
O2	0.034 (2)	0.042 (3)	0.042 (2)	0.0081 (19)	0.0009 (17)	−0.013 (2)
C1	0.026 (2)	0.033 (3)	0.029 (2)	0.004 (2)	0.0043 (19)	0.003 (2)
C2	0.028 (3)	0.027 (3)	0.025 (3)	−0.0007 (19)	0.002 (2)	0.0008 (19)
C3	0.026 (2)	0.030 (3)	0.032 (3)	0.003 (2)	0.0063 (19)	0.005 (2)
C4	0.032 (2)	0.028 (3)	0.032 (3)	0.005 (2)	0.009 (2)	0.000 (2)
C5	0.035 (3)	0.050 (4)	0.027 (3)	0.008 (3)	0.000 (2)	−0.009 (3)
C6	0.032 (3)	0.050 (3)	0.034 (3)	0.012 (3)	−0.005 (2)	−0.009 (3)
C7	0.029 (3)	0.032 (3)	0.029 (3)	0.005 (2)	0.004 (2)	0.001 (2)
C8	0.037 (3)	0.025 (2)	0.030 (3)	0.002 (2)	0.014 (2)	0.003 (2)

### Geometric parameters (Å, °)

**Table:**

Pb1—O2	2.366 (4)	N2—C7	1.375 (7)
Pb1—O2^i^	2.366 (4)	O1—C8	1.251 (8)
Pb1—O1^i^	2.395 (5)	O2—C8	1.259 (8)
Pb1—O1	2.395 (5)	C2—C3	1.382 (8)
Pb1—C8	2.749 (6)	C2—C7	1.406 (8)
Pb1—C8^i^	2.749 (6)	C3—C4	1.399 (8)
Pb1—S1^ii^	3.3894 (17)	C3—H3	0.9300
Pb1—S1^iii^	3.3894 (17)	C4—C5	1.404 (8)
S1—C2	1.741 (6)	C4—C8	1.489 (8)
S1—C1	1.776 (5)	C5—C6	1.378 (9)
N1—C1	1.330 (8)	C5—H5	0.9300
N1—H1A	0.8600	C6—C7	1.392 (9)
N1—H1B	0.8600	C6—H6	0.9300
N2—C1	1.310 (7)		
			
O2—Pb1—O2^i^	102.8 (3)	H1A—N1—H1B	120.0
O2—Pb1—O1^i^	80.64 (17)	C1—N2—C7	110.9 (5)
O2^i^—Pb1—O1^i^	54.26 (15)	C8—O1—Pb1	92.4 (4)
O2—Pb1—O1	54.26 (15)	C8—O2—Pb1	93.6 (4)
O2^i^—Pb1—O1	80.64 (17)	N2—C1—N1	125.0 (5)
O1^i^—Pb1—O1	106.4 (3)	N2—C1—S1	114.6 (4)
O2—Pb1—C8	27.21 (17)	N1—C1—S1	120.4 (4)
O2^i^—Pb1—C8	92.19 (17)	C3—C2—C7	122.5 (5)
O1^i^—Pb1—C8	94.21 (19)	C3—C2—S1	129.0 (5)
O1—Pb1—C8	27.04 (17)	C7—C2—S1	108.5 (4)
O2—Pb1—C8^i^	92.19 (17)	C2—C3—C4	117.7 (5)
O2^i^—Pb1—C8^i^	27.21 (17)	C2—C3—H3	121.2
O1^i^—Pb1—C8^i^	27.04 (17)	C4—C3—H3	121.2
O1—Pb1—C8^i^	94.21 (19)	C3—C4—C5	120.6 (5)
C8—Pb1—C8^i^	93.9 (2)	C3—C4—C8	119.7 (5)
O2—Pb1—S1^ii^	132.35 (10)	C5—C4—C8	119.7 (5)
O2^i^—Pb1—S1^ii^	69.61 (11)	C6—C5—C4	120.5 (6)
O1^i^—Pb1—S1^ii^	121.04 (10)	C6—C5—H5	119.7
O1—Pb1—S1^ii^	78.27 (11)	C4—C5—H5	119.7
C8—Pb1—S1^ii^	105.21 (14)	C5—C6—C7	120.1 (6)
C8^i^—Pb1—S1^ii^	95.37 (14)	C5—C6—H6	120.0
O2—Pb1—S1^iii^	69.61 (11)	C7—C6—H6	120.0
O2^i^—Pb1—S1^iii^	132.35 (11)	N2—C7—C6	124.8 (6)
O1^i^—Pb1—S1^iii^	78.27 (11)	N2—C7—C2	116.7 (6)
O1—Pb1—S1^iii^	121.04 (10)	C6—C7—C2	118.6 (6)
C8—Pb1—S1^iii^	95.37 (14)	O1—C8—O2	119.7 (5)
C8^i^—Pb1—S1^iii^	105.21 (14)	O1—C8—C4	120.8 (6)
S1^ii^—Pb1—S1^iii^	149.76 (6)	O2—C8—C4	119.5 (6)
C2—S1—C1	89.4 (3)	O1—C8—Pb1	60.5 (3)
C1—N1—H1A	120.0	O2—C8—Pb1	59.2 (3)
C1—N1—H1B	120.0	C4—C8—Pb1	178.7 (5)

Symmetry codes: (i) −*x*+1, *y*, −*z*+3/2; (ii) *x*, −*y*, *z*+1/2; (iii) −*x*+1, −*y*, −*z*+1.

### Hydrogen-bond geometry (Å, °)

**Table:**

*D*—H···*A*	*D*—H	H···*A*	*D*···*A*	*D*—H···*A*
N1—H1B···O1^iv^	0.86	2.11	2.973 (7)	179
N1—H1A···N2^v^	0.86	2.09	2.934 (7)	168

Symmetry codes: (iv) *x*, −*y*−1, *z*−1/2; (v) −*x*+2, −*y*−2, −*z*+1.
